# Microbiological safety of a novel bio-artificial liver support system based on porcine hepatocytes: a experimental study

**DOI:** 10.1186/2047-783X-17-13

**Published:** 2012-05-25

**Authors:** Bing Han, Xiao-lei Shi, Yue Zhang, Xue-hui Chu, Jin-yang Gu, Jiang-qiang Xiao, Hao-zhen Ren, Jia-jun Tan, Zhong-ze Gu, Yi-tao Ding

**Affiliations:** 1 Department of Hepatobiliary Surgery, the Affiliated DrumTower Hospital of Nanjing University Medical School, Number 321 Zhongshan Road, Nanjing 210008, China; 2 Department of Hepatobiliary Surgery, DrumTower Clinical Medical College of Nanjing Medical University, Number 321 Zhongshan Road, Nanjing 210008, China; 3 State Key Laboratory of Bioelectronics, Southeast University, Nanjing 210008, China

**Keywords:** Microbiological safety, Porcine endogenous retrovirus, Bio-artificial liver, Porcine hepatocyte, Mesenchymal stem cells

## Abstract

**Background:**

Our institute has developed a novel bio-artificial liver (BAL) support system, based on a multi-layer radial-flow bioreactor carrying porcine hepatocytes and mesenchymal stem cells. It has been shown that porcine hepatocytes are capable of carrying infectious porcine endogenous retroviruses (PERVs) into human cells, thus the microbiological safety of any such system must be confirmed before clinical trials can be performed. In this study, we focused on assessing the status of PERV infection in beagles treated with the novel BAL.

**Methods:**

Five normal beagles were treated with the novel BAL for 6 hours. The study was conducted for 6 months, during which plasma was collected from the BAL and whole blood from the beagles at regular intervals. DNA and RNA in both the collected peripheral blood mononuclear cells (PBMCs) and plasma samples were extracted for conventional PCR and reverse transcriptase (RT)-PCR with PERV-specific primers and the porcine-specific primer *Sus scrofa* cytochrome B. Meanwhile, the RT activity and the *in vitro* infectivity of the plasma were measured.

**Results:**

Positive PERV RNA and RT activity were detected only in the plasma samples taken from the third circuit of the BAL system. All other samples including PBMCs and other plasma samples were negative for PERV RNA, PERV DNA, and RT activity. In the *in vitro* infection experiment, no infection was found in HEK293 cells treated with plasma.

**Conclusions:**

No infective PERV was detected in the experimental animals, thus the novel BAL had a reliable microbiological safety profile.

## Background

Acute liver failure (ALF) is a serious clinical disease with high mortality rate. Although liver transplantation is currently recognized as the most effective treatment for ALF, its application has been seriously limited by the lack of donor organs, the high cost of treatment, and the requirement for life-long immunosuppressive therapy [1,2]. Therefore, bio-artificial liver (BAL) support systems, based on functional hepatocytes, have received extensive attention because of their unique biological function, and considerable progress has been made in their development [3-5]. Currently, porcine hepatocytes are still the main cell sources for such systems because of their adequate resources, accessibility, and features similar to human hepatocytes [6-8]. However, because they are xenogeneic cells, porcine hepatocytes are associated with a number of problems, including that of microbiological safety. Porcine endogenous retrovirus (PERV) was first discovered in 1971 in porcine kidney (PK15) cells [9]. In 1997, Patience *et al*. showed for the first time that PERV released from PK15 could infect human embryonic kidney (HEK)293 cells *in vitro*[10]. Subsequently, PERV-A and PERV-B were confirmed as the human-tropic subtypes [11]. It was later found that PERV could successfully infect a variety of human cells *in vitro*, including endothelial cells, fibroblasts and bone marrow stromal cells, and virus replication was seen in some of these cells. Two other *in vivo* studies showed that implanted porcine islets could result in PERV infection in non-obese diabetic/severe combined immunodeficiency (NOD/SCID) mice [12]. Thus, transmission of PERV is a microbiological safety issue that cannot be ignored in BAL systems using porcine hepatocytes.

We developed a novel multi-layer radial-flow bioreactor containing galactosylated chitosan nanofiber scaffolds, which we found to have a high level o fefficiency *in vitro*[13]. The bioreactor is composed of a stack of 65-layer round flat plates and a cylindrical container with an inlet on the top and an outlet on the bottom. A co-culture system of porcine hepatocytes and mesenchymal stem cells (MSCs), which had been established previously in our institute [14], was perfused into the bioreactor to act as functional cells, and a new extracorporeal BAL support system based on the novel multi-layer radial-flow bioreactor and the co-culture system of porcine hepatocytes and MSCs. was constructed. This study is a preclinical experiment identifying the microbiological safety of the novel BAL. In this study, we used five normal dogs to assess transmission of PERV. The aim of this research was to investigate whether there was a possibility of transmission of PERV into the experimental dogs using this new BAL support system.

## Methods

### Animals and reagents

All animal procedures were performed in accordance with institutional and national guidelines and with the approval by the Animal Care Ethics Committee of Nanjing University and Nanjing Drum Tower Hospital.

Five outbred white pigs with an average weight of 15–20 kg and five inbred beagles with an average weight of 11–13 kg were used for the experiments.

All cell culture-related reagents were purchased from Gibco (Grand island, N.Y.USA).

### Establishing co-culture system of porcine hepatocytes and mesenchymal stem cells

A co-culture system of porcine hepatocytes and MSCs was prepared as described previously [14]. In brief, bone marrow was aspirated from the iliac crest of the pigs, then the mononuclear cells were collected over a Ficoll histopaque layer by gradient centrifugation (20 minutes, 400 g, density 1.077 g/ml) and seeded at a density of 1 × 10^6^ cells/cm^2^ in growth medium containing low-glucose DMEM supplemented with 10% FBS, 100 IU/ml penicillin and 100 mg/ml streptomycin. The culture medium was replaced after the first 24 hours, and the subculture was prepared according to standard cell-culture techniques. The cultured cells were confirmed as MSCs by analysis (FACScan; Becton Dickinson, San Jose, CA, USA) of surface markers, including CD45, CD29, CD44, and CD90. The primary pig hepatocytes were harvested using a two-step *in situ* collagenase perfusion technique. The viability of the isolated primary hepatocytes, as determined by trypan blue exclusion, was greater than 95%. Non-parenchymal cells were identified based on size (<10 μm in diameter) and morphology (nonpolygonal or stellate), and made up less than 1% of cells, which was confirmed by immunocytochemical analysis of albumin and cytokeratin 18. The mixed suspension of fresh hepatocytes and MSCs during passages 3 to 5 (2:1) was perfused at a density of 10^6^ cells/ml into a substratum of 500 ml RPMI-1640 without sera, and incubated in our new bioreactor at 37°C and 5% CO_2_.

### Construction and application of the new bio-artificial liver support system

**Figure 1:**
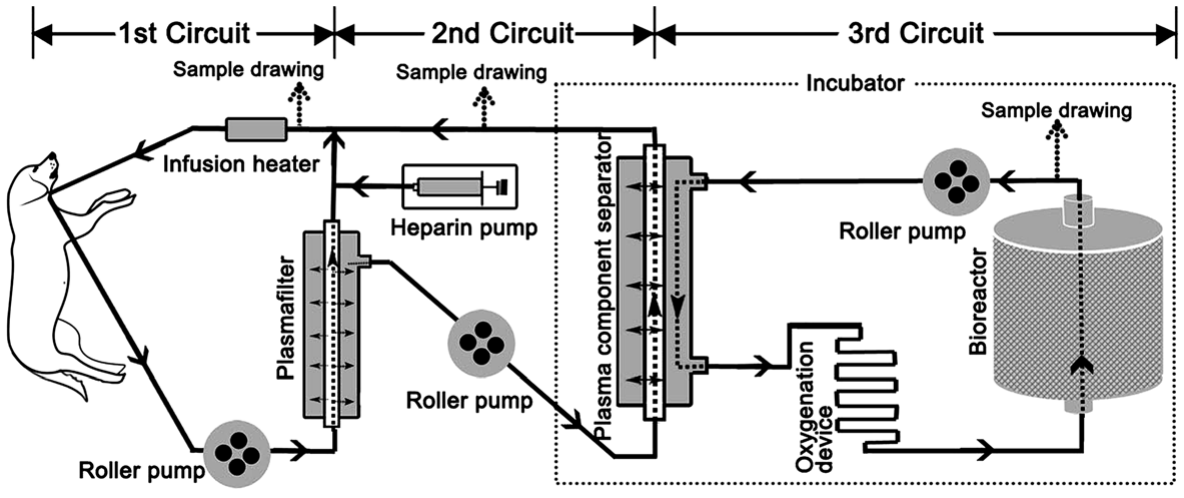
**Construction of the bio-artificial liver support system.** The system consists of three roller pumps, a heparin pump, an infusion heater, a plasmafilter, a plasma component separator, an oxygenation device, and a multi-layer radial-flow bioreactor containing galactosylated chitosan nanofiber scaffolds. The bioreactor and the oxygenation device is kept in an incubator with the internal temperature of 37°C. All devices are connected by sterile tubing. The dotted arrow indicates where the samples were drawn into the circuits.

The new BAL support system consisted of three roller pumps, a heparin pump, an infusion heater, a plasma filter (Sorin Group Italia, Mirandola, Italy), a plasma component separator (Kawasumi Laboratories Inc, Tokyo, Japan) serving as immunoprotective barrier, an oxygenation device, and a multi-layer radial-flow bioreactor containing galactosylated chitosan nanofiber scaffolds. The bioreactor and the oxygenation device was prepared and kept in an incubator with am internal temperature of 37°C as previously reported [13]. The whole system was then assembled as shown in Figure 1.

Catheters were inserted into the internal carotid artery and internal jugular vein of the dogs under continued anesthesia by intravenous administration of propofol (Diprivan; Astrazeneca, Wuxi, China) at a dose of 10 mg/kg/h, and the catheters were then connected to the BAL device (Figure 1). The BAL treatment was begun 4 hours after the cells were seeded, when most cells were adhered to the galactosylated chitosan nanofiber scaffolds. During the first circuit, the whole blood in was perfused at a rate of 40 ml/min for 6 hours, then during the second circuit, the plasma was separated from the plasma filter at a rate of 15 ml/min, and finally during the third circuit, the plasma was filtered through the plasma component separator at a rate of 15 ml/min. Heparin (100U/kg) was administered intravenously to the dogs at the start of treatment and continued at a dose of 40 U/kg/h into the first circuit, and removed 30 minutes before the end of the treatment. Samples of the plasma circulating in the BAL system and samples of whole blood from the beagles were collected at regular intervals (before treatment, at 3 and 6 hours during treatment, and at 12 hours, 1, 3, 5 and 7 days, 2, 3, and 4 weeks, and 3 and 6 months after treatment) until the research was completed 6 months after the treatment. Complete blood count (CBC) was assessed for each blood sample. PBMCs and plasma were separated from the whole blood, and frozen at −80°C for later use.

### DNA extraction and PCR

Total DNA was extracted from the plasma and the separated PBMCs by means of a DNA extraction kit (Axygen Scientific Inc.,Union City, CA, USA) in accordance with the manufacturer’s instructions, using the primers shown in Table 1[15,16]. PCR conditions were 50°C for 30 minutes, then 95°C for 5 minutes, followed by 35 cycles of 94°C for 30 seconds, 55°C for 45 seconds, 72°C for 30 seconds, with a final extension step of 72°C for 5 minutes. Samples were separated by gel electrophoresis in 2% agarose gels and stained with ethidium bromide. DNA and RNA extracted from PK15 cells was used as positive control and pure water as negative control.

**Table 1:** Primers used for PCR

**Name**	**Direction**	**Sequence 5′→3′**
Protease-specific	Forward	GCTACAACCATTAGGAAAACTAAAAG
	Reverse	AACCAGGACTGTATATCTTGATCAG
Polymerase-specific	Forward	CTACAACCATTAGGAAAACTAAAAG
	Reverse	AACCAGGACTGTATATCTTGATCAG
SsCytB	Forward	CATTGGAGTAGTCCTACTATTTACCG
	Reverse	GTAGGATTAGTATTATAAATAAGGCTCCT
β-actin	Forward	GCTCGTCGTCGACAACGGCTC
	Reverse	CAAACATGATCTGGGTCATCTTCTC

SsCytB, *Sus scrofa* cytochrome B.

### RNA extraction and reverse transcriptase PCR

Total RNA was extracted from the PBMCs and the plasma with Trizol reagent (Invitrogen Inc., Carlsbad, USA), and dissolved in diethyl pyrocarbonate-treated water in accordance with the manufacturer’s instructions. The extracted RNAs had an OD 260/280 of 1.60 and 2.00, respectively. DNAs were reverse-transcribed to cDNA using commercial kits (Biouniquer Technology Co.,Ltd, Hongzhou, China) in accordance with the manufacturer’s instructions, then PCR was performed as described above.

### Reverse transcriptase activity assay

The RT activity of the plasma was assessed using a commercial kit (C-type RT activity Kit; Cavidi-Tech, Uppsala, Sweden), in accordance with the manufacturer’s instructions.

### Exposure of canine peripheral blood mononuclear cells to porcine endogenous retrovirus

PERV was harvested from the supernatant of PK15 cells and concentrated by sucrose density-gradient centrifugation [17]. Canine PBMCs isolated from the blood of normal dogs were exposed to PERV in a culture medium composed of 1 ml PERV and 4 ml of high-glucose DMEM with 0.8 g/ml polybrene (hexadimethrine bromide; Sigma Aldrich) for 24 hours. The cells were then washed with PBS twice, and subcultured for 1 month. Finally, the cells were collected for assessment of PERV by PCR, RT-PCR and RT activity assay. Previously. The infected cells were used as a positive control.

### *In vitro* infection experiments

The *in vitro* infection experiments were performed as described previously [18], with some modifications HEK-293 cells (gift from Professor Hua, Nanjing University) were passaged overnight in 25-ml tissue culture flasks and then incubated in a culture composed of 2 ml of separated plasma harvested at defined times, and 3 ml of DMEM-HG with 0.8 g/ml polybrene. Meanwhile, the supernatant of PK15 cells and 0.8 g/ml polybrene was inoculated into the culture of HEK293 cells as a positive control. After 4 hours of exposure at 37°C, the inoculum was removed, and the cell monolayer was washed twice with PBS. The cells were then cultured with high-glucose DMEM supplemented with 10% FBS, and passaged to confluence for 1 month before collection. The collected HEK293 cells were tested for PERV DNA by PCR. RT activity was measured in the supernatant of treated HEK293 cells.

## Results

### Status of animals receiving bio-artificial liver treatment

Animals had their heart and respiratory rates monitored continuously by an electrocardiography, and these were stable during the extracorporeal perfusion. All dogs were able to stand and take food within 12 hours from the end of the treatment., and their biological behavior recovered to the pre-treatment state within 72 hours. All animals survived throughout the entire 6-month study.

### Complete blood count

The results of the CBC are listed in Table 2.

**Table 2:** Complete blood count of dogs with bio-artificial liver treatment

**Time points**	**White blood cells, × 10^9^/l**	**Red blood cells, × 10^12^/l**	**Platelets, × 10^12^/l**
Before treatment	13.0 ± 4.72	5.6 ± 1.27	252.1 ± 100.22
After 3 hours of treatment	9.2 ± 9.56	6.9 ± 1.44	159.9 ± 106.28
6 h during treatment	33.7 ± 6.20	7.2 ± 0.76	244.0 ± 76.47
1 day after treatment	29.4 ± 12.16	7.2 ± 0.98	229.72 ± 99.63
3 day after treatment	17.9 ± 8.04	5.6 ± 1.16	205.3 ± 161.58
7 day after treatment	17.3 ± 5.36	4.6 ± 1.18	214.3 ± 159.55

### Detection of porcine endogenous retrovirus DNA and RNA in peripheral blood mononuclear cells

**Figure 2:**
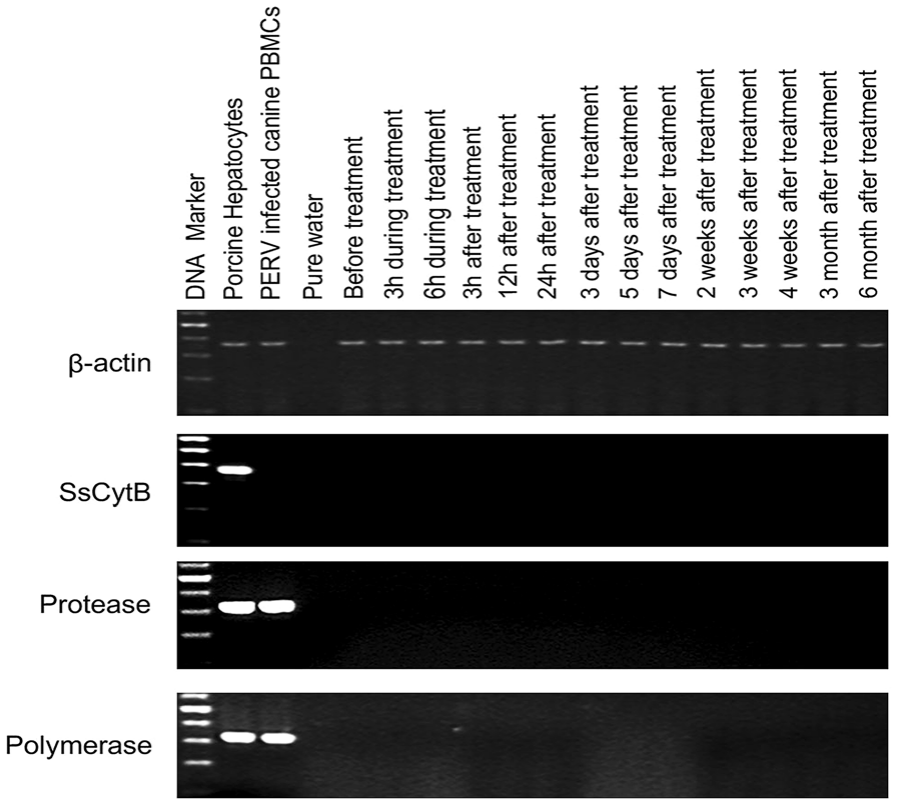
**Representative results of PCR electrophoresis with the DNA of beagle peripheral blood mononuclear cells (PBMCs).** No PERV DNA or *Sus scrofa* cytochrome B (SsCytB) sequence was found in the canine PBMCs. DNA from porcine hepatocytes and PERV-infected canine peripheral blood mononuclear cell (PBMCs) were used as positive controls, and pure water was used as the negative control. Ladder ranged from 100 to 600 bp.

The agarose gel electrophoresis results showed that there was no PERV DNA or RNA in the dog PBMCs collected at various times. Screening for the porcine-specific gene SsCytB did not find this gen in the DNA of the PBMCs of any of the five dogs (Figure 2).

### Detection of porcine endogenous retrovirus DNA and RNA in plasma

**Figure 3:**
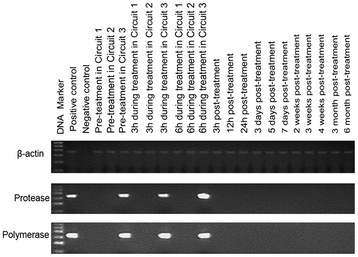
**Representative results of reverse transcriptase PCR electrophoresis with the RNA extracted from the plasma.** The protease and polymerase gene were found only in circuit 3 before and during treatment. C+, PK15 cells; C–, pure water. The ladder ranged from 100 to 600 bp.

Except for positive bands representing the protease and polymerase gene in circuit 3 before and during treatment, all results of RT-PCR with the RNA from collected plasma were negative (Figure 3). The same results were obtained for plasma DNA, and no SsCytB sequence was seen in the plasma.

### Reverse transcriptase activity assay

The RT activity was examined twice for all collected plasma samples. The results showed that the RT activity was limited to the plasma in the third circuit before and at 3 hours of treatment. RT activity was not detected in any the other samples including all dog plasma collected during and after the treatment (Table 3).

**Table 3:** Reverse transcriptasde activity of the plasma at defined time intervals

**Time point**	**Beagle**				
	**1**	**2**	**3**	**4**	**5**
Pre-treatment in circuit 3	Positive*	Positive*	Positive*	Positive*	Positive*
After 3 hours of treatment in circuit 3	Positive*	Negative	Negative	Positive	Negative
After 6 hours of treatment in circuit 3	Negative	Negative	Negative	Negative	Negative
Pre-treatment in circuit 2	Negative	Negative	Negative	Negative	Negative
After 3 hours of treatment in circuit 2	Negative	Negative	Negative	Negative	Negative
After 6 hours of treatment in circuit 2	Negative	Negative	Negative	Negative	Negative
Pre-treatment in circuit 1	Negative	Negative	Negative	Negative	Negative
After 3 hours of treatment in circuit 1	Negative	Negative	Negative	Negative	Negative
After 6 hours of treatment in circuit 1	Negative	Negative	Negative	Negative	Negative
Pre-treatment in circuit 1	Negative	Negative	Negative	Negative	Negative
3 hours after treatment	Negative	Negative	Negative	Negative	Negative
12 hours after treatment	Negative	Negative	Negative	Negative	Negative
24 hours after treatment	Negative	Negative	Negative	Negative	Negative
3 days after treatment	Negative	Negative	Negative	Negative	Negative
5 days after treatment	Negative	Negative	Negative	Negative	Negative
7 days after treatment	Negative	Negative	Negative	Negative	Negative
2 weeks after treatment	Negative	Negative	Negative	Negative	Negative
1 month after treatment	Negative	Negative	Negative	Negative	Negative
3 months after treatment	Negative	Negative	Negative	Negative	Negative
6 months after treatment	Negative	Negative	Negative	Negative	Negative

*Positive result.

### Infection of HEK293 cells *in vitro*

Results were negative for PERV-specific genes including protease and polymerase genes and the porcine-specific SsCytB sequence in the DNA from HEK293 cells inoculated with collected plasma. DNA extracted from PK15-infected HEK293 cells and the pure water was used as positive control and negative control, respectively. All supernatants from treated HEK293 cells were negative for RT activity.

## Discussion

We report the development of a novel BAL system based on a new multi-layer radial-flow bioreactor containing galactosylated chitosan nanofiber scaffolds and a co-system of porcine hepatocytes and MSCs. This was developed in our institute because clinical trials of other BAL systems have shown disappointing results [3,4]. To improve the cellular function, each plate was covered with nanofiber scaffolds to mimic the topography of extracellular matrix (ECM), and the galactose was grafted onto the nanofibers to mimic the biochemical environment of ECM [13]. We used a co-culture system of porcine hepatocytes and MSCs at a ratio of 2:1, which was introduced into the BAL system [14]. Both of these two elements have been shown to have better hepatoctye-specific function *in vitro*, so we expected our BAL to be superior to previous devices. A plasma component separator was placed between circuits 2 and 3 to allow with media exchange by passive diffusion across the semipermeable membrane inside, as with other BAL systems [4].

PERV is known to exist generally in the porcine genome. Various porcine cells can excrete PERV particulates, which have been shown to infect a variety of human cells *in vitro*[12], and a transient PERV infection in guinea pigs was seen *in vivo*[19]. Previous reports claimed infection in severe combined immunodeficiency mice [20,21] and nude mice [22] after injection of porcine islet cells. Pseudotyping with murine endogenous retroviruses has been shown to be the mechanism of transmission [23,24]. It was found that dogs possess the same huPAR-1 and muPAR gene sequence coding the PERV receptors in humans, and were able to express functional receptors for PERV, which implied that dogs might be infected by PERV through the same mechanism as humans [25-27].

Reviewing the studies on the microbiological safety of BALs, we found no evidence of crossspecies transmission of PERV in patients treated with porcine BALs or living pig tissue to date [18,28-37]. In previous studies, we did not find any obvious enhanced PERV expression in freshly isolated porcine hepatocytes by chitosan nanofiber scaffold [38,39]. Although a number of studies have investigated the possibility of PERV transmission, no clear conclusion has been drawn. Therefore, viral security is one of the most important issues that must be resolved before our novel extracorporeal BAL system can be used for clinical experiments.

PCR and RT-PCR are the most common methods used to detect PERV transmission [12], and thus were used in the present study. We assessed several pairs of primers reported in previous publications [15,16,40], using them in a number of PCR and RT-PCR assays with nucleic acid extracted from canine cells. The three sets of primers finally used (protease, polymerase, and SsCytB) were chosen because of their high specificity for PERV detection in canine cells (data not shown), and the sensitivity of PCR assays with these three primers has been reported as 0.25 of a PK-15 cell per 10^4^ cells [16], 0.3 of a PK-15 cell per 10^5^ cells [29], and 0.25 of a porcine cell per 10^5^ cells [16], respectively.

In the current study, PERV RNA, PERV DNA, and reactive RT were detected only in plasma samples taken during circuit 3 of treatment. No activity was found in plasma during circuits 1 or 2, or in PBMCs collected from the dogs. All assays were negative for the porcine-specific SsCytB sequence. These results indicated that there was a possibility of infectious PERV being in the system, but confined to circuit 3. For detection of microchimerism, we carried out PCR with the porcine-specific SsCytB primers [16]. The results were negative for PERV and SsCytB genes, suggesting absence of microchimerism. Furthermore, negative RT activity, consistent with the absence of PERV RNA in the animal plasma, indicated no infection of the animals by PERV, as RT activity is a generic marker for retroviruses [41]. These results are similar to those of Kuddus *et al*., who reported that the RT activity and PERV RNA were limited to the shell of the bioreactor in their bio-artificial liver support system (BLSS) [30]. In the plasma in circuit 3, we found positive RT activity in all five samples before treatment and in two of the five samples after 3 hours of treatment, but in none of the five samples after 6 hours of treatment. This eventual absence of RT activity may result from the dilution effect of plasma on the reverse transcriptase or from an inhibiting effect of the animal plasma on the RT activity.

An important part of our BAL system is the plasma component separator, which uses semipermeable membranes of 10 nm, far smaller than the approximately 100 nm diameter of the retrovirus [42]. Furthermore, the duration of blood perfusion was just 6 hours, thus it is reasonable to infer that the PERV DNA and RNA were removed by the filtration from the collected plasma and PBMCs of the beagles in our study, and thus that no PERV was transmitted across the plasma component separator during the treatment phase. We considered that the lack of PERV transmission in our system is likely to be the result of a combination of no direct contact between the porcine hepatocytes and the dog blood, the small pore size of the semipermeable membranes in the plasma component separator, and the short term of BAL treatment.

We also performed i*n vitro* infection experiments to assess the infectivity of PERV in our system and the infection state of the dogs. Nyberg *et al*. [43] reported that HEK293 cells could not be infected by the supernatant of *in vitro* cultured porcine hepatocytes, and that human plasma from patients with fulminant hepatic failure would not affect its infectivity. In other trials, no HEK293 cells were found to be infected in an *in vitro* infection experiment of another two extracorporeal BAL systems, the Academic Medical Center (AMC)-BAL and the HepatAssist system [18,28]. Similarly, we found no infection of HEK293 cells by PERV in our system.

In conclusion, we found no evidence of PERV infection in our dog model after treatment with our novel BAL support system, suggesting that the system does not pose a risk for PERV transmission. These results indicate that the microbiological safety profile of the system should be sufficient to allow clinical trials. However, BAL treatment of patients would be a more complicated process. Further studies into the microbiological safety of our BAL system in clinical used need to be performed.

## Abbreviations

BAL: Bio-artificial liver support system; CBC: complete blood count; DMEM: Dulbecco’s modified Eagle’s medium; ECM: Extracellular matrix; FBS: fetal bovine serum; MSC: Mesenchymal stem cells; PBMC: Peripheral blood mononuclear cell; PBS: Phosphate-buffered saline; PERV: Porcine endogenous retrovirus.

## Competing interests

The authors declare that they have no competing interests.

## Authors’ contributions

Han B conceived of the study, participated in its design, carried out the virological detection, and coordination and helped to draft the manuscript. Zhang Y, Chu XH, Gu JY carried out the animal experiment, participated in the sequence alignment and drafted the manuscript. Xiao JQ, and Tan JJ were responsible for cell culture. Shi XL participated in the design of the study and performed the statistical analysis. Gu ZZ and Ding YT provided new reagents/analytic tools. All authors read and approved the final manuscript.
